# Soluble Starch Synthase III-1 in Amylopectin Metabolism of Banana Fruit: Characterization, Expression, Enzyme Activity, and Functional Analyses

**DOI:** 10.3389/fpls.2017.00454

**Published:** 2017-03-30

**Authors:** Hongxia Miao, Peiguang Sun, Qing Liu, Caihong Jia, Juhua Liu, Wei Hu, Zhiqiang Jin, Biyu Xu

**Affiliations:** ^1^Key Laboratory of Tropical Crop Biotechnology, Ministry of Agriculture, Institute of Tropical Bioscience and Biotechnology, Chinese Academy of Tropical Agricultural SciencesHaikou, China; ^2^Key Laboratory of Genetic Improvement of Bananas, Hainan Province, Haikou Experimental Station, Chinese Academy of Tropical Agricultural SciencesHaikou, China; ^3^Commonwealth Scientific and Industrial Research Organization Agriculture and FoodCanberra, ACT, Australia

**Keywords:** banana (*Musa acuminata* L.), soluble starch synthase, amylopectin metabolism, expression analysis, SSIII-1 function

## Abstract

Soluble starch synthase (SS) is one of the key enzymes involved in amylopectin biosynthesis in plants. However, no information is currently available about this gene family in the important fruit crop banana. Herein, we characterized the function of MaSSIII-1 in amylopectin metabolism of banana fruit and described the putative role of the other MaSS family members. Firstly, starch granules, starch and amylopectin content were found to increase during banana fruit development, but decline during storage. The SS activity started to increase later than amylopectin and starch content. Secondly, four putative *SS* genes were cloned and characterized from banana fruit. Among them, *MaSSIII-1* showed the highest expression in banana pulp during fruit development at transcriptional levels. Further Western blot analysis suggested that the protein was gradually increased during banana fruit development, but drastically reduced during storage. This expression pattern was highly consistent with changes in starch granules, amylopectin content, and SS activity at the late phase of banana fruit development. Lastly, overexpression of *MaSSIII-1* in tomato plants distinctly changed the morphology of starch granules and significantly increased the total starch accumulation, amylopectin content, and SS activity at mature-green stage in comparison to wild-type. The findings demonstrated that *MaSSIII-1* is a key gene expressed in banana fruit and responsible for the active amylopectin biosynthesis, this is the first report in a fresh fruit species. Such a finding may enable the development of molecular markers for banana breeding and genetic improvement of nutritional value and functional properties of banana fruit.

## Introduction

Starch is the most widespread carbohydrate storage molecule in plants and plays a vital role in human nutrition, food industry and chemical manufacturing. The physicochemical properties of starch are strongly affected by its two key components, amylose and amylopectin (Zeeman et al., 2010; Bischof et al., 2013). In higher plants, there are at least six classes of enzymes that are involved in amylose and amylopectin biosynthesis: ADP-Glc pyrophosphorylase (AGPase), granule-bound starch synthase (GBSS), soluble starch synthase (SS), starch branching enzyme (SBE), starch debranching enzyme (DBE), and starch phosphorylase (SP) (Fujita et al., 2008; Szydlowski et al., 2009; Jeon et al., 2010; Subasinghe et al., 2014).

Starch synthase uses ADP-glucose for chain elongation *via* α-1,4-glycosidic linkages, and directly catalyzes the amylopectin biosynthesis (Szydlowski et al., 2009, 2011). A plant typically carries at least four SS subfamilies, termed as SSI, II, III, and IV, respectively (Dian et al., 2005). Comparison of the deduced amino acid sequences showed that these four subfamilies are highly similar in a span of approximately 450 amino acid residues in the C-terminus that comprises the catalytic and starch-binding domains (Schwarte et al., 2013). The sizes of SSI, II, and III proteins were estimated to be approximately 67, 81, and 112 kDa, respectively, in Arabidopsis leaves (Delvallé et al., 2005; Zhang et al., 2008; Gámez-Arjona et al., 2014). *SS* gene expression was recently profiled in a numbers of plants including rice (Crofts et al., 2015), maize (Liu et al., 2015; Huang et al., 2016), potato (Edwards et al., 1999), Arabidopsis (Schwarte et al., 2013), wheat and taro (Li et al., 2000; Lin and Jeang, 2005). However, different SSs may predominate in storage organs of different species e.g., SSI in rice endosperm, SSII in wheat (Li et al., 2000; Crofts et al., 2015). In common wheat, *SSIII* expression was detected from very early to the middle stages of endosperm development (Li et al., 2000). Rice *SSIII-2* and *SSIV-1* showed the highest expression level at the middle and late development stages of rice endosperm, respectively (Dian et al., 2005).

Amylopectin chain length distribution and amylopectin/ amylase ratio affected starch functionality (Dian et al., 2005; Zhang et al., 2005). Biochemical and gene overexpression experiments revealed that SSI, II, and III are involved in the elongation of short (dp 8–12) (Fujita et al., 2008), medium (dp 13–25), and long (dp > 30) starch chains (Szydlowski et al., 2011; Zhu et al., 2014), respectively. SSIV is closely related to SSIII; both have similar structures (Leterrier et al., 2008). Further reverse genetic studies showed that impaired *SSI* expression was reported to result in structurally altered amylopectin in Arabidopsis leaves (Delvallé et al., 2005) and rice endosperm (Takemoto-Kuno et al., 2006; Fujita et al., 2008). Inhibition of *SSII* gene expression gave rise to lower pasting temperature as the result of alterations in amylopectin structure (Edwards et al., 1999). Loss of *SSIII* expression reduced the proportion of amylopectin with very long chains and affected the amylopectin/amylase ratio in maize (Zhu et al., 2014), Arabidopsis (Zhang et al., 2005), and rice (Dian et al., 2005). A defective mutant of *SSIV* displayed a severely compromised growth phenotype with fewer but larger starch granules within the plastid in Arabidopsis (Roldán et al., 2007). However, Zhang et al. (2008) reported that some SSs, such as SSII and SSIII, may overlap in amylopectin biosynthesis in Arabidopsis. In addition, GBSSI from other SS isoforms also contributed to amylopectin synthesis in rice and *Chlamydomonas reinhardtii* (Ral et al., 2006; Hanashiro et al., 2008). These evidences suggested the important roles of the *SS* genes in regulating plant amylopectin metabolism.

Banana (*Musa* spp.) is not only one of the most highly consumed fruits in the world but also widely used as a staple food in the tropical and subtropical regions (D’Hont et al., 2012). The typical starch content of a green dessert banana fruit accounts for 20∼25% of fresh weight or 60∼75% of dry weight (DW), and the starch granules are relatively large (8∼48 μm in diameter) compared to those of cereals (Hubbard et al., 1990; de Barros Mesquita et al., 2016). Hence banana is also a potential excellent model plant for studying fresh fruit starch metabolism. Current research on banana starch has mainly focused on granule structure (Low et al., 2015) and antioxidant capacities (Sarawong et al., 2014), as well as physicochemical properties of starch (Zhang and Hamaker, 2012; Utrilla-Coello et al., 2014). Several key genes involved in starch biosynthesis or conversion of starch to sucrose have been isolated and characterized in banana, including *MaGBSSI* (Miao et al., 2014), *DBE* (Bierhals et al., 2004), *SUCROSE PHOSPHATE SYNTHASE* (*SPS*), *SUCROSE SYNTHASE* (*SuSy*), and *INVERTASE* (Hubbard et al., 1990). However, to our knowledge, the function of *SS* genes has not been characterized in banana.

Amylopectin is the major component of immature banana fruit (Torre-Gutiérrez et al., 2008), which has a number of remarkable features, including higher retrogradation and lower pasting temperature (Sarawong et al., 2014), relative to those in rice (Syahariza et al., 2013), maize (Huang et al., 2016), and potato (Wikman et al., 2014). Due to the important role of SS in amylopectin metabolism, there is a need to investigate the function of SS in banana. In this study, we revealed that *MaSSIII-1* is a key gene expressed in banana fruit and responsible for the active amylopectin biosynthesis by expression, enzyme activity, and functional analyses. This result could be used in the genetic manipulation of banana fruit for genetic improvement of its nutritional values as well as value-added industrial applications, such as high starch foods, higher retrogradation and lower pasting temperature raw processing materials.

## Materials and Methods

### Plant Materials and Treatments

Banana (*M. acuminata* L. AAA group, *cv.* ‘Dwarf Cavendish’; ITC 0002) seedlings were obtained from the banana tissue culture center (Chinese Academy of Tropical Agricultural Sciences, Danzhou, China) and were grown at 28°C with 70% humidity, 200 μmol⋅m^-2^⋅s^-1^ light intensity, and long day condition (16 h light/8 h dark cycle). When banana seedlings produced five leaves, they were planted in the Institute of Tropical Bioscience and Biotechnology banana plantation (Chengmai, Hainan, 20N, 110E) until harvest. Roots, stems, leaves, bracts, flowers, peels, and pulps at 60 days after emergence from the pseudostem (DAF) were collected separately using tweezers, frozen immediately in liquid nitrogen, and stored at -80°C until expression analysis in different tissues. For each biological replicate, two banana hands having a similar developmental stage from two plants were selected and six fingers from the hands were obtained. Banana pulps at 0, 10, 20, 30, 40, 50, and 60 DAF were collected, immediately frozen in liquid nitrogen, and stored at -80°C until starch/amylopectin quantification, mRNA and protein expression analysis.

Banana hands (60 DAF) were separated into individual fingers representing the same developmental stage. For natural ripening treatment, banana samples were kept at 22°C and allowed to ripen in open air. In accordance with the previously published banana ripening stages (Miao et al., 2014), fruits stored for 0, 5, 10, 15, 20, 25, and 30 days period of post-harvest (DPH) were frozen in liquid nitrogen and stored at -80°C until starch/amylopectin quantification, mRNA and protein expression analysis.

### Scanning Electron Microscopy (SEM) Observation

Pulp samples were fixed in stubs using double-faced tape and coated with a 10 nm thick platinum layer using a Bal-tec MED 020 Coating system (Kettleshulme, UK) before analysis with a FEI Quanta 600 FEG Scanning Electron Microscope (FEI Company, Hillsboro, OR, USA). SEM observations were performed using the secondary electron mode operating at 15 kV.

### Determination of Total Starch Content, Amylopectin Content, and SS Activity

Banana pulp was immersed in 0.5% (w/v) sodium bisulfite solution for 10 min to prevent browning, and then dried at 40°C for 20∼24 h. The dried pulp was milled to powder and suspended in 5 mL 80% (w/v) Ca(NO_3_)_2_, placed in a boiling water bath for 10 min, and centrifuged for 4 min at low speed (3,800 g). The supernatant was then transferred to a 20 mL volumetric flask. The total starch, amylose, and amylopectin contents of the extract were determined following Yang et al. (1992) and Miao et al. (2014). The amylopectin content was calculated by subtracting the amylose content from the total starch content. Enzymatic analysis of SS activity was carried out following Nakamura et al. (1989). A unit represents increasing 0.01 OD value per min at 340 nm.

### Cloning and Sequence Analysis of Genes Encoding *SS* in Banana Fruit

RNA purification and cDNA synthesis were performed as previously described by Li et al. (2015). To obtain full-length cDNAs, sequences of four *SS* family members, including*SSI* (GSMUA_Achr3G03290_00), *SSII* (GSMUA_Achr6G23190_001), *SSIII-1* (GSMUA_Achr11G18570_001), and *SSIII-2* (GSMUA_Achr5G00700_001), were retrieved from the banana DH-Pang (AA group) genome sequence database^1^. Four primer pairs (**Supplementary Table S1**) were designed based on these sequences. The *SS* coding sequences were submitted to BLAST analysis to recover their corresponding genomic DNA sequences. Exon lengths were calculated by alignment of genomic DNA sequences with cDNA sequences, and introns were determined according to the “GC-AG” rule (Miao et al., 2014). The deduced amino acid sequences were aligned using Clustal W, and a phylogenetic tree was constructed based on the neighbor-joining (NJ) method with a Kimura 2-parameter model using MEGA5.0 software (Arizona State University, Tempe, AZ, USA). The numerical value for each interior branch is the percent bootstrap value calculated from 1,000 replicates.

### Quantitative Real-Time PCR (qRT-PCR) Analysis

Specific primer pairs were designed using Primer 5.0 software. Primers that had high specificity and efficiency on the basis of melting curve analysis were used to conduct quantification analysis (**Supplementary Table S1**). Moreover, PCR products were sequenced to confirm the specificity of primer pairs. Amplification efficiencies of primer pairs ranged from 0.9 to 1.1. The levels of *MaSS* and *SISSIII-1* expression were quantified by qRT-PCR using an iQ5 real-time PCR detection system (Bio-Rad, Hercules, CA, USA) with the SYBR *Ex*Script RT-PCR Kit (TaKaRa, Dalian, China). *ACTIN* or *GAPDH* that were verified to be constitutive in expression and hence suitable to be used as internal controls were used as reference genes to normalize transcriptional levels of each *MaSS* gene and *SISSIII-1* (**Supplementary Table S1**). Relative expression levels of four *MaSS* genes were analyzed in three technical replicates and calculated using the 2^-ΔΔ^*^C^*^T^ method (Livak and Schmittgen, 2001). Each sample contains three biological replicates.

### Detection of MaSSIII-1 Protein Levels by Western Blot Analysis

Pulp samples (0.5 g) were homogenized with a set of mortar and pestle on ice in an equal volume of solution, which consisted of 50 mM HEPES-NaOH (pH 7.4), 2 mM MgCl_2_, 50 mM β-mercaptoethanol, and 12.5% (v/v) glycerol (Nishi et al., 2001). A 20 μL aliquot of the supernatant was incubated with 36 μL of solution I consisting of 50 mM HEPES-NaOH (pH 7.4), 1.6 mM adenosine diphosphate glucose (ADPG), 0.7 mg amylopectin, and 15 mM DL-dithiothreitol (DTT) for 20 min at 30°C. Following the reaction termination at 100°C for 30 s, 20 μL solution II was added, which consisted of 50 mM HEPES-NaOH, 4 mM phosphoenolpyruvate (PEP), 200 mM KCl, 10 mM MgCl_2_, and 1.2 U pyruvate kinase, and incubation on ice for 5 min. The reaction was incubated at 30°C for 20 min before it was terminated at 100°C for 30 s. A 60 μL aliquot of the supernatant was mixed with 43 μL of solution III (50 mM HEPES-NaOH, 10 mM glucose, 20 mM MgCl_2_, 2 mM nicotinamide adenine dinucleotide phosphate, 1.4 U hexokinase, 0.35 U glucose-6-phosphate dehydrogenase) and incubated at 30°C for 10 min. The supernatant (SS enzyme solution) was used to Western blot analysis.

For Western blot analysis, 30 μL of the SS enzyme solution from banana pulps at different developmental stages extracted by the above methods was loaded on each lane and separated on a 12% polyacrylamide gel. Proteins were then transferred onto Hybond^TM^-N^+^ membranes (Amersham Biosciences, Buckinghamshire, UK). Membranes were probed with rabbit anti-MaSSIII-1 polyclonal antibody (Abmart, Shanghai, China) and banana actin antibody (control) in 1:1,000 dilution in PBS-Tween 20 plus 3% BSA, respectively, followed by alkaline phosphatase-conjugated anti-rabbit IgG secondary antibody (Sigma, St. Louis, MO, USA) in 1:1,000 dilution. Positive signals on the membranes were detected with a 5-bromo-4-chloro-3-indolyl-phosphate/nitro blue tetrazolium (BCIP/NBT) solution (Amresco, Solon, OH, USA).

### Plant Transformation, Generation and Southern Blot Analysis of *MaSSIII-1* Transgenic Plants

The entire *MaSSIII-1* coding region was inserted into the pCAMBIA-1302 vector under the transcriptional control of CaMV 35S promoter following a double digestion with *Nco* I and *Spe* I. The pCAMBIA-1302-MaSSIII-1 was transferred into *A. tumefaciens* strain LBA4404. Transgenic tomato (*Solanum lycopersicum* L.) plants were generated using the Agrobacterium-mediated transformation as previously described (Arshad et al., 2014). Kanamycin-resistant transgenic tomato lines were selected and the transgene integration was determined by Southern blot analysis. Genomic DNA (10 μg per sample) from transgenic tomato leaves was isolated using a CTAB method (Li et al., 2015) and digested with *Eco*R I overnight at 37°C, separated on a 0.8% (w/v) agarose gel and transferred onto Hybond-N^+^ nylon membranes (Hybond N^+^, Amersham, UK) (Li et al., 2015). Probes were prepared from the PCR product amplified using the primers (5′-gagagagaagatggtggaatctat-3′ and 5′-aggagcactagaccagtcatgac-3′) and labeled with DIG-dUTP according to the manufacturer’s instructions (Roche Applied Science, Mannheim, Germany). Fruits from two single-copy transgenic plants L4 and L11 were used for expression analysis and functional investigation of MaSSIII-1 in comparison to wild-type.

### Statistical Analysis

Three biological replicates were performed for each sample, unless specified otherwise. Statistical analyses were performed using Microsoft Excel and SPSS (Chicago, IL, USA). Analysis of variance was used to compare the statistical difference based on Student’s *t*-tests, at significant levels of *p* < 0.05 (^∗^), and *p* < 0.01 (^∗∗^).

## Results

### Change in Starch Granules, Total Starch Content, Amylopectin Content, and SS Enzyme Activity at Different Developmental and Storage Stages of Banana Fruit

**FIGURE 1:**
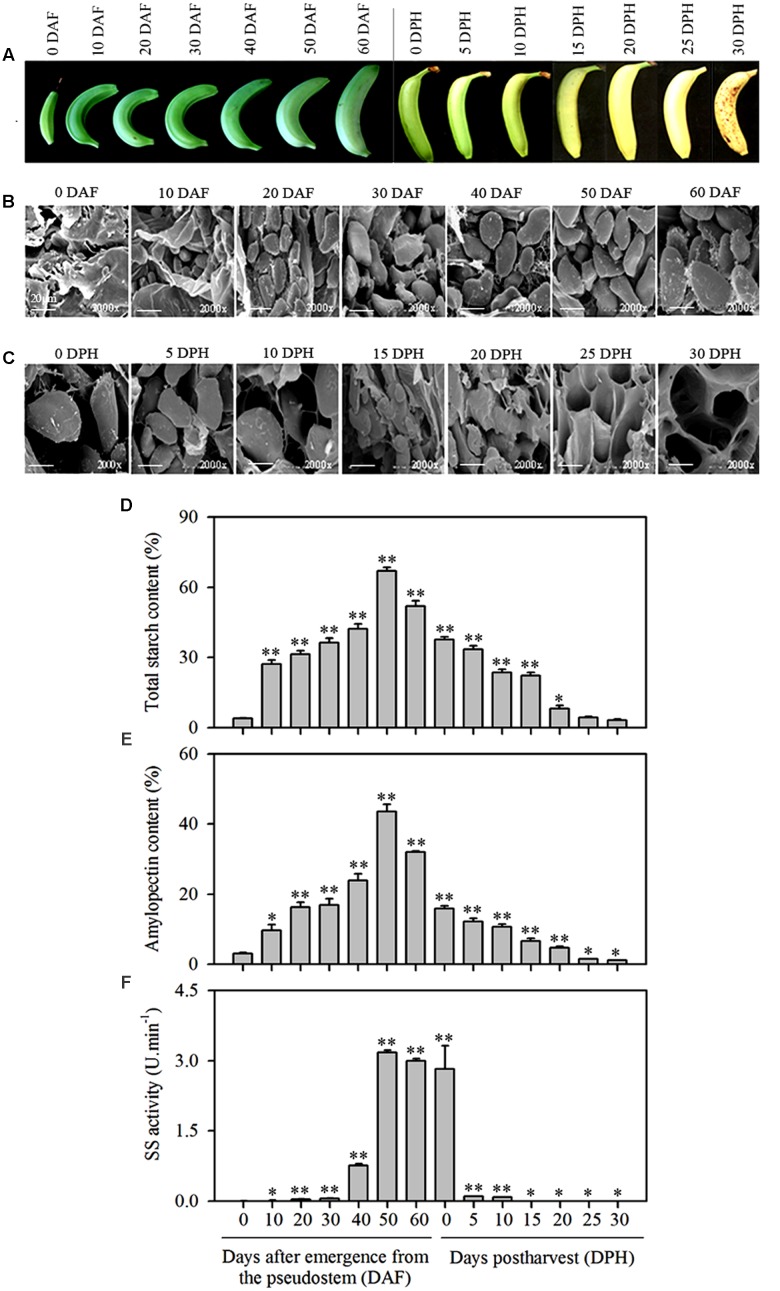
**Changes in starch granules, total starch content, amylopectin content, and SS activity in banana pulp at different stages of development and during storage.** **(A)** Banana fruit at different development stages (0, 10, 20, 30, 40, 50, and 60 DAF) and following storage for varying amounts of time (0, 5, 10, 15, 20, 25, and 30 DPH), **(B)** SEM of starch granules in pulp during banana fruit development, **(C)** SEM of starch granules in pulp during banana fruit storage, **(D)** Total starch content in banana pulp at different stages of development and during storage, **(E)** Amylopectin content in banana pulp at different stages of development and during storage, **(F)** SS activity in banana pulp at different stages of development and during storage. DAF: days after emergence from the pseudostem; DPH: days of post-harvest. The vertical bars represent the mean ± SD of three replicates. Asterisks indicate significant difference from 0 DAF and 0 DPH vs. the following days (^∗^*p* < 0.05; ^∗∗^*p* < 0.01). Scale bar = 20 μm.

During banana fruit development the fruit size gradually increases and it becomes curled in a crescent shape as fruit develops (**Figure 1A**). As observed using SEM, starch granules in banana pulp were barely detectable at the initial sampling time, i.e., 0 DAF, but the oval-shaped starch granules become clearly visible and were significantly increased in size as the fruit developed from 10 (6.0∼8.2 μm) to 60 DAF (28.0∼35.7 μm). Morphological comparison of starch granules during this period of time indicated that the shape and number of starch granules remained consistent, but the size was significantly increased and maximized at 60 DAF (**Figure 1B**).

Starch granules in mature banana pulp were also observed using SEM at the 30 DPH during storage at 22°C, at 5 days’ intervals. Compared to the first time point (0 DPH), the quantity of starch granules per unit volume decreased rapidly over time. After 15 DPH of storage, starch granules were hardly detectable (**Figure 1C**).

The total starch content and amylopectin content in pulp increased as fruit developed and peaked at 50 DAF (these two polymers reached to 67 and 44% DW, respectively) before decline at the 60 DAF time point and continued to decrease gradually during storage (**Figures 1D,E**), implying that after maturity starch and amylopectin may have experienced a rapid degradation process. However, the SS enzyme activity increased later than starch and amylopectin accumulation at maturity and had an abrupt decline at the very beginning of storage (**Figure 1F**).

### Nucleotide Sequence Characteristics, Chromosomal Localization, and Phylogenetic Analysis of Banana *MaSS* Genes

**FIGURE 2:**
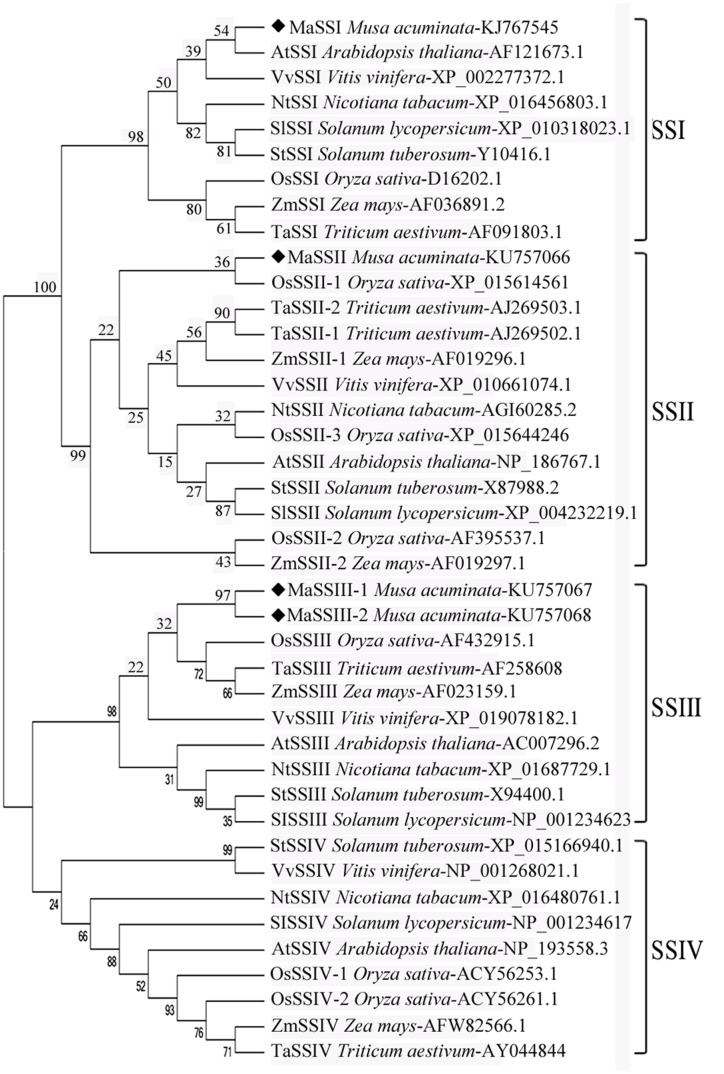
**Phylogeny of MaSSI, MaSSII, MaSSIII-1, and MaSSIII-2 amino acid sequences in relation to other known SS sequences.** Bootstrapping was performed with 1000 replicates. The numbers indicated for each clade represent bootstrap support values given as percentages. Scale bar represents 0.2 substitutions per amino acid position.

Full-length cDNAs encoding *MaSSI*, *MaSSII*, *MaSSIII-1*, and *MaSSIII-2* were 2,076 bp, 1,851 bp, 2,397 bp, and 3,267 bp, respectively. All these sequences were deposited at GenBank and their accession numbers were listed in **Figure 2**. BLAST analysis against the banana DH-Pang (AA group) genome sequence database^2^ revealed that *MaSSI*, *MaSSII*, *MaSSIII-1*, and *MaSSIII-2* are located on chromosome 3, 6, 11, and 5, respectively (**Supplementary Figure S1**). The sequence analysis also revealed markedly different primary structures as *MaSSI*, *MaSSII*, *MaSSIII-1*, and *MaSSIII-2* contain 25, 6, 5, and 9 exons, respectively. The stop codon usage is also different as TAA was used by *MaSSI* and *MaSSIII-1*, but TGA was used by *MaSSII* and *MaSSIII-2* (**Supplementary Figure S1**).

The deduced amino acid sequences of the MaSSI and MaSSII shared three conserved regions, referred to as Domain I, II, and III (**Supplementary Table S2**), as previously identified in SS enzymes from amaranth (Park et al., 2012). The MaSSIII-1 and MaSSIII-2 amino acid sequences contained four distinct regions: a transit peptide region, a variable repeat region, a SSIII specific region, and a C-terminal catalytic domain (**Supplementary Table S3**), as previously identified in SSIII enzymes from wheat (Li et al., 2000). According to pI/MW software analysis^3^, the predicted molecular weights of the MaSSI, MaSSII, MaSSIII-1, and MaSSIII-2 proteins were 76.69, 69.85, 90.69, and 119.26 kDa, respectively, and their theoretical pIs were 5.24, 5.36, 6.44, and 8.21, respectively.

To elucidate phylogenetic relationship of banana *MaSS* genes, the four deduced polypeptide sequences were aligned with orthologous SS sequences and a NJ tree was constructed (**Figure 2**). MaSSIII-1 and MaSSIII-2 are aligned next to each other, forming a side branch in association with other SSIII sequences from monocot species. Similarly, MaSSI and MaSSII are clustered together with their respective orthologs from other species. This may also indicate that the divergence of the SSI, SSII and SSIII occurred prior to the speciation of these plant species, but the separation of SSIII-1 and SSIII-2 might be of a more recent history likely through a gene duplication and subsequent divergence.

### Spatially and Temporally Differential Expression of Four *MaSS* Genes

**FIGURE 3:**
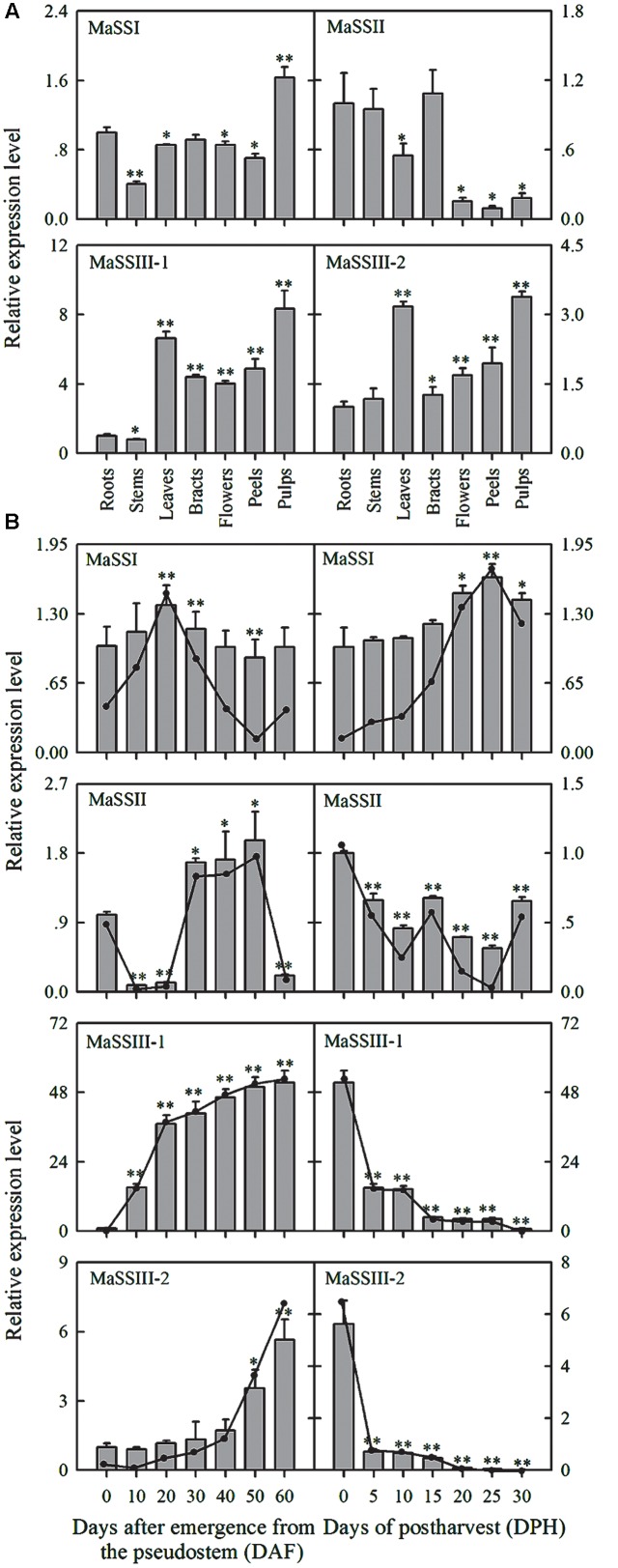
**Expression of *MaSSI*, *MaSSII*, *MaSSIII-1*, and *MaSSIII-2* genes in different banana tissues** **(A)** and in fruit at different stages of development and after various storage times **(B)**. The *y*-axis represents the relative fold-difference in mRNA level, which was calculated using the 2^-ΔΔCt^ formula with *MaActin* and *MaGAPDH* as internal controls. The vertical bars represent the mean ± SD of three replicates. Asterisks indicate significant difference from 0 DAF and 0 DPH vs. the following days (^∗^*p* < 0.05; ^∗∗^*p* < 0.01).

The spatial expression patterns of the four *MaSS* genes were analyzed by qRT-PCR in seven different tissues, including roots, stems, leaves, bracts, flowers, peels, and pulp (60 DAF) (**Figure 3A**). *MaSSI* was expressed at low levels in all tissues examined with the highest expression in pulps. *MaSSII* was also found to express low in all the vegetative tissues, including roots, stems, leaves, and bracts, and particularly low in flowers and fruit tissues. The expression patterns of *MaSSIII-1* and *MaSSIII-2* were very similar, with highest expression in pulps and leaves (starch content in leaves was approximately 22.67 ± 1.32%), but that *MaSSIII-1* had the highest levels of expression.

The temporal expression of *MaSSI*, *MaSSII*, *MaSSIII-1*, and *MaSSIII-2* during banana fruit development was also determined by qRT-PCR (**Figure 3B**). Despite of being highly variable, the expression levels of *MaSSI* and *MaSSII* genes were found to be generally low throughout the entire banana pulp development. Both *MaSSIII-1* and *MaSSIII-2* showed a sharp increase of expression during the fruit development, the increase in expression occurred earlier in *MaSSIII-1* than in *MaSSIII-2*, with the highest expression detected at the last sampling stage when banana had reached its maturity. However, the expression of *MaSSIII-1* was about 10∼20-fold higher than that of *MaSSIII-2* during the fruit development.

When the naturally ripen banana fruits were subjected to varying period of storage, the *MaSS* genes were also found to be differentially expressed (**Figure 3B**). *MaSSII* showed consistently low levels of expression throughout the storage period, but the *MaSSI* expression was slightly increased during storage. The seemingly negative association between *MaSSI* expression and starch degradation needs to be further investigated. Although there can be a lack of congruency between transcript and activity, it is possible that there can be some starch biosynthesis occurring even during net degradation (Luengwilai and Beckles, 2009). In contrast, a drastic reduction of expression was detected at 5 days after storage, as observed in both *MaSSIII-1* and *MaSSIII-2*.

### Western Blot Analyses of MaSSIII-1 Protein

**FIGURE 4:**
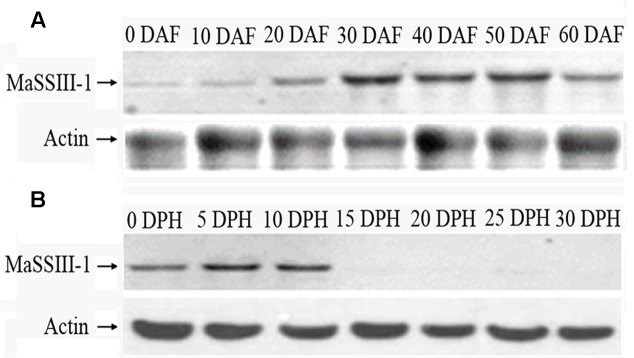
**Western blot analyses of the MaSSIII-1 protein.** **(A)** Western blot analyses of MaSSIII-1 proteins in banana pulp at different stages of development, **(B)** Western blot analyses of MaSSIII-1 proteins in banana pulp at different stages of storage. DAF: days after emergence from the pseudostem; DPH: days of post-harvest.

Western blot analysis with rabbit anti-MaSSIII-1 polyclonal antibody as a probe indicated that the size of MaSSIII-1 is approximately 90.0 kDa, which is consistent with the molecular weight (90.69 kDa) as predicted by the PeptideMass program. The expression of MaSSIII-1 protein was gradually increased during banana fruit development, but drastically reduced from 0 to 30 DPH of storage (**Figures 4A,B**). The Western blot results was consistent with changes in starch granules (**Figures 1B,C**), amylopectin content (**Figure 1E**) and SS activity (**Figure 1F**) during banana development and ripening, suggesting the expression of MaSSIII-1 protein might play an important role in regulating amylopectin metabolism in banana fruit during development and ripening.

### Overexpression of *MaSSIII-1* in Tomato Changes the Morphology of Starch Granules and Increases the Amylopectin Content and SS Activity

**FIGURE 5:**
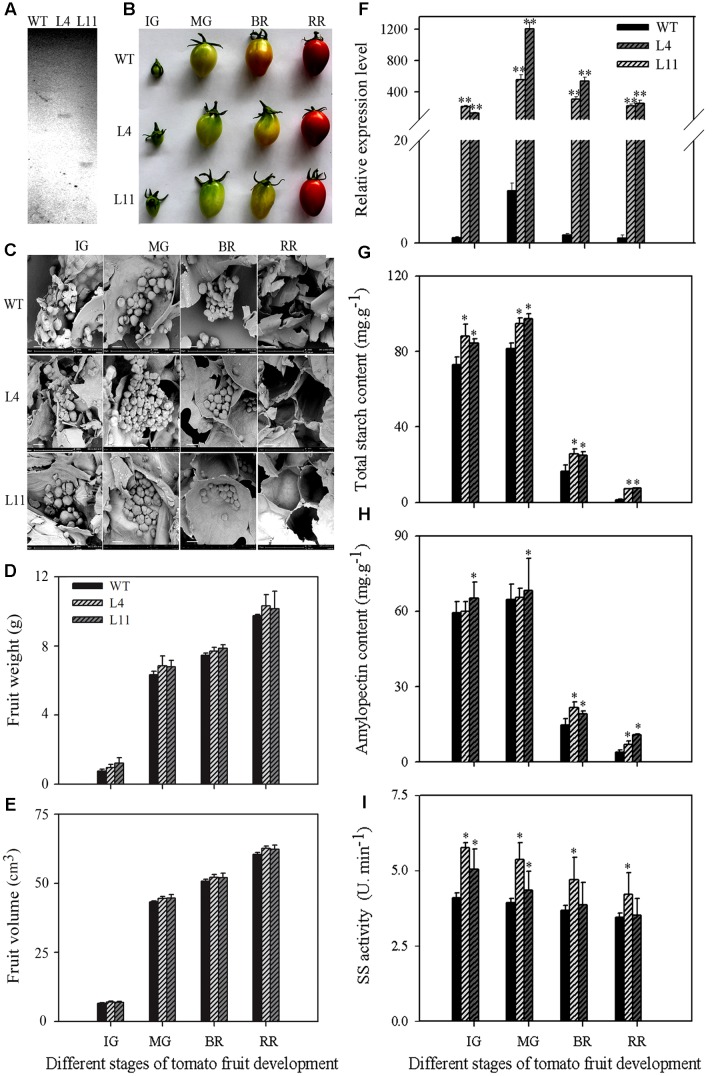
**Southern blot analysis** **(A)** and change of fruit shape **(B)**, starch granule **(C)**, fruit weight **(D)**, fruit volume **(E)**, gene expression **(F)**, total starch content **(G)**, amylopectin content **(H)**, and SS enzyme activity **(I)** at different developmental stages of *MaSSIII-1* transgenic plants in tomato. WT: wild-type; L4, L11: *MaSSIII-1* transgenic plants; IG: immature green; MG: mature-green; BR: orange-breaker; RR: red ripening stage. Asterisks indicate significant difference between the WT and *MaSSIII-1* transgenic plants (^∗^*p* < 0.05; ^∗∗^*p* < 0.01). Three biological experiments were performed, which produced similar results. Scale bar = 15 μm.

To further examine the function of *MaSSIII-1* during fruit development and ripening, *MaSSIII-1* was introduced into a pCAMBIA-1302 vector under the transcriptional control of the CaMV 35S promoter. Two single-copy transgenic plants (named L4 and L11) were identified by Southern blot analysis (**Figure 5A**). The fruit shape, starch granule morphology, gene expression, total starch content, amylopectin content, and SS activity were investigated in the wild-type (WT) and the *MaSSIII-1* overexpressing transgenic plants.

Compared to WT, the fruit shape of *MaSSIII-1* transgenic plants did not change (**Figure 5B**), but the morphology of starch granules was significantly altered from immature green (IG) to orange-breaker (BR) in transgenic tomato plants, severe cracks in the surface of starch granules were observed (**Figure 5C**). For fruit weight and volume, there are no significant differences between *MaSSIII-1* transgenic plants and WT (**Figures 5D,E**). Overexpression of *MaSSIII-1* in tomato plants significantly increased the gene expression (**Figure 5F**), *SISSIII-1* (NP_001234623; *MaSSIII-1* orthologs) expression in tomato fruit was lower than that of *MaSSIII-1* transgenic plants (**Figure 5F** and **Supplementary Figure S2**). At IG and mature-green (MG) stages, total starch content, amylopectin content, and SS activity showed significant differences between transgenic plants and WT (**Figures 5G–I**). Especially at the MG stage, the gene expression, total starch content, amylopectin content, and SS activity significantly increased 55∼120-fold, 13.47∼15.83 mg⋅g^-1^, 6.36∼9.17 mg⋅g^-1^, and 0.54∼0.80 U⋅min^-1^ in the transgenic fruits compared to WT, respectively.

## Discussion

Despite of the extensive studies on cereal starch, little information is available regarding the dynamics of starch accumulation (net result of starch synthesis and degradation), SS activity, and amylopectin metabolism during fruit development and storage in fresh starchy fruits, such as banana. In tomato, starch is transiently accumulated during fruit development and degraded within the lifecycle of that organ, while starch synthesis and degradation are occurring simultaneously (Luengwilai and Beckles, 2009). However, change characteristics of SS activity are unclear. In this study, a temporal modulation in starch content was found concomitant to banana fruit development and storage. The result was consistent with the report of tomato (Luengwilai and Beckles, 2009). SS activity increased as the starch granule expanded in size and amylopectin content increased during fruit development, but decreased significantly along with the degradation of amylopectin and starch granule during storage. Moreover, we found that SS activity increased later than starch and amylopectin accumulation, the same was true for transcript and protein analyses, implying SS act at later stages of fruit development while other enzymes may act in the early phases.

The SS isoforms comprise at least four families (namely SSI, SSII, SSIII, and SSIV) in plants and play important roles in amylopectin biosynthesis (Park et al., 2012). GBSS as a separate from the other SS isoforms also influences biosynthesis of extra-long unit chains of amylopectin in rice (Hanashiro et al., 2008). Six *GBSS* genes have been cloned and identified in banana (Miao et al., 2014). In this report, we characterized the function of MaSSIII-1 in amylopectin metabolism of banana fruit and described the putative role of the other MaSS family members. Four MaSSs could be categorized into three classes, including MaSSI, MaSSII, and MaSSIIIs (MaSSIII-1 and MaSSIII-2). This scenario seems to apply in some lower green algae species where the gene distribution of SSIV is patchy (Deschamps et al., 2008). Moreover, in Arabidopsis the role of SSIV in starch granule seeding can be replaced, in part, by the phylogenetically related SSIII (Szydlowski et al., 2011). Sequence analysis indicated that the *MaSSI*, *II*, *III-1*, and *III-2* genes showed characteristics of a typical plant *SS* gene (Li et al., 2000; Park et al., 2012). The MaSSI and MaSSII amino acid sequences shared three conserved regions encoding Domain I, II, and III, respectively (Park et al., 2012). Domain I is a putative N-terminal transit peptide, Domains II and III are the C-terminal catalytic domain (Park et al., 2012). The amino acid sequences of MaSSIII-1 and MaSSIII-2 contained four distinct regions: a transit peptide region, a variable repeat region, a SSIII specific region, and a C-terminal catalytic domain. The SSIII domain organization was reported in wheat (Li et al., 2000).

Starch synthase paralogs show varying spatial-temporal expression (Senoura et al., 2004; Dian et al., 2005). *MaSSI* and *MaSSII* were found to express in low levels in vegetative organs, flower and banana fruits, similar to their homologs in kidney bean (Senoura et al., 2004) and rice (Jiang et al., 2004). In comparison, *MaSSIII-1* and *MaSSIII-2* were abundantly expressed in the late phase of developing fruit pulp, with the expression level of *MaSSIII-1* being significantly higher (**Figure 3A**). Such an observation is consistent with previous studies in rice, wheat, and Arabidopsis (Li et al., 2000; Dian et al., 2005; Busi et al., 2008; Valdez et al., 2008). Taken together, it is proposed that the four *MaSS* genes cloned from banana fruit may play divergent roles, with *MaSSI* and *MaSSII* being house-keeping, but *MaSSIII-1* and *MaSSIII-2* expression at transcription level clearly occurs at later stages of fruit development, they’re not involved in amylopectin synthesis during early phase of fruit development.

SSIII protein as a catalytic factor plays an important role in amylopectin metabolism (Zhu et al., 2014; Huang et al., 2016). SSIII contributes the major activity (80% of the total) in potato tubers (Abel et al., 1996). In Arabidopsis, loss of both SSII and SSIII caused slower plant growth and dramatically reduced starch content (Zhang et al., 2008). Defective *SSIII* mutations resulted in change of starch structure into large clusters with more singly branched building blocks in amylopectin in maize (Zhu et al., 2014). Inhibition of *SSIII* expression resulted in a drastic drop in the overall rate of starch accumulation and the proportion of amylopectin with very long chains in potato (Abel et al., 1996; Edwards et al., 1999). SSIII activity was found to affect starch phosphorylation in potato tubers (Carpenter et al., 2015). In fresh fruits, whether starch metabolism was affected by SS, as far as we know there has not been reported. In this study, MaSSIII-1 transcript in both transgenic lines was 100-fold higher than WT. SS activity, total starch content, and amylopectin content were enhanced significantly by overexpressing *MaSSIII-1* gene in tomato transgenic plants at MG stage (**Figure 5**), suggesting these parameter increases may be attributed to MaSSIII because of increased transcript and polypeptide. Similar results were reported in wheat (Li et al., 2000) and rice (Dian et al., 2005), but this is the first report in a fresh fruit species. In addition, we found that overexpression of *MaSSIII-1* in tomato fruit showed severe cracks in the surface of starch granules and changed the starch granules morphology. This study suggests that *MaSSIII-1* is a key gene in the amylopectin biosynthesis and therefore it could be used as a useful tool for marker assisted molecular breeding in banana.

## Conclusion

Starch synthase activity increased along with the amylopectin accumulation at later stages of banana fruit development, but declined during storage. Four *SS* genes encoding *MaSSI*, *MaSSII*, *MaSSIII-1*, and *MaSSIII-2* were cloned and characterized. Expression pattern of only MaSSIII-1 was highly consistent with dynamic changes in starch granules, amylopectin content, and SS activity. *MaSSIII-1* transgenic lines distinctly changed the morphology of starch granules. Overexpression of *MaSSIII-1* in tomato plants significantly increased the amylopectin accumulation and SS activity in comparison to WT. This is the first report about the *MaSSIII-1* gene involved in amylopectin metabolism in a fresh fruit species. These findings establish a solid foundation to further regulate the amylopectin metabolism in banana fruits or other fresh fruits using the *MaSSIII-1* or its homologous genes.

## Author Contributions

ZJ and BX conceived and designed the experiments. HM, PS, QL, CJ, JL, and WH performed the experiments and carried out the analysis. HM, PS, and QL designed the experiments and wrote the manuscript. All authors read and approved the final manuscript.

## Conflict of Interest Statement

ZJ and BX conceived and designed the experiments. HM, PS, QL, CJ, JL, and WH performed the experiments and carried out the analysis. HM, PS, and QL designed the experiments and wrote the manuscript. All authors read and approved the final manuscript.

## Footnotes

1 http://banana-genome.cirad.fr/

2 http://banana-genome.cirad.fr/

3 http://expasy.org/computepi/

## Supplementary Material

**FIGURE S1 | Sequence motifs and chromosomal localization of four *MaSS* genes in banana.** **(A)** Structural organization of banana *MaSS* genes, solid boxes indicate exons, and bold lines represent introns, **(B)** Chromosomal localization of banana *MaSS* genes.

**FIGURE S2 | Expression of *SISSIII-1* at different stages of development in tomato fruit.** IG: immature green; MG: mature-green; BR: orange-breaker; RR: red ripening stage. The *y*-axis represents the relative fold-difference in mRNA level, which was calculated using the 2^-ΔΔCt^ formula. The vertical bars represent the mean ± SD of three replicates. Asterisks indicate significant difference from IG stage vs. the following stages (^∗^*p* < 0.05; ^∗∗^*p* < 0.01).

**TABLE S1 | Primers uxsed in this study.**

**TABLE S2 | Comparison of the deduced amino acid sequences of banana fruit MaSSI and MaSSII.** The three consensus regions (Domain I, II, and III) for both starch synthases are highlighted in bold and box.

**TABLE S3 | Comparison of the deduced amino acid sequences of banana fruit MaSSIII-1 and MaSSIII-2.** The four consensus regions (Transit peptide region, Variable repeat region, SSIII specific region, and Catalytic domain) for both starch synthases are highlighted in bold and box.
