# Supplementary figures and images for: Soluble Starch Synthase III-1 in Amylopectin Metabolism of Banana Fruit: Characterization, Expression, Enzyme Activity, and Functional Analyses
Source: Front Plant Sci. 2017 Mar 30;8:454. doi: 10.3389/fpls.2017.00454 (PMC5371607; doi:10.3389/fpls.2017.00454)

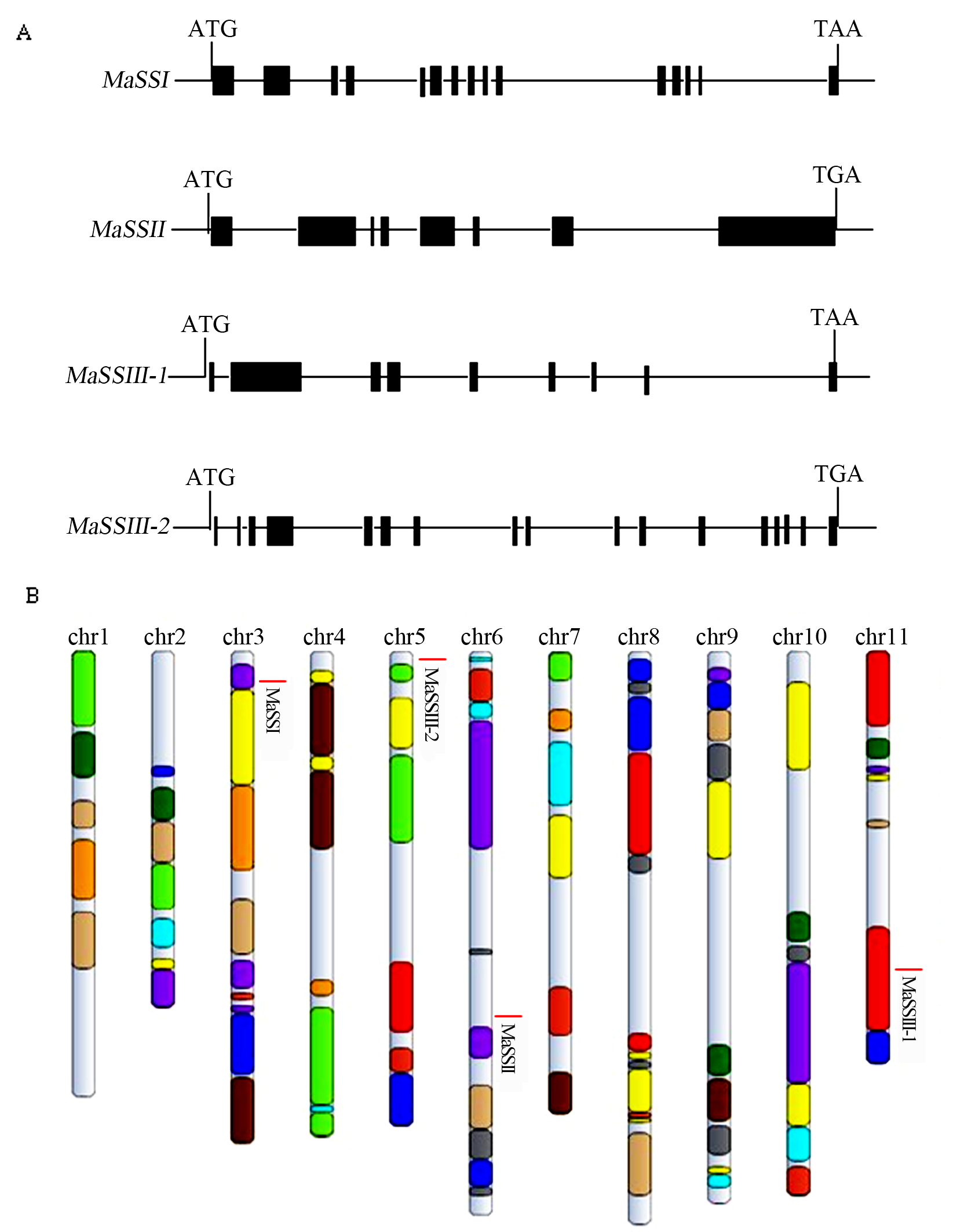

Supplement: Supplementary file 1 [file Image_1.TIFF]

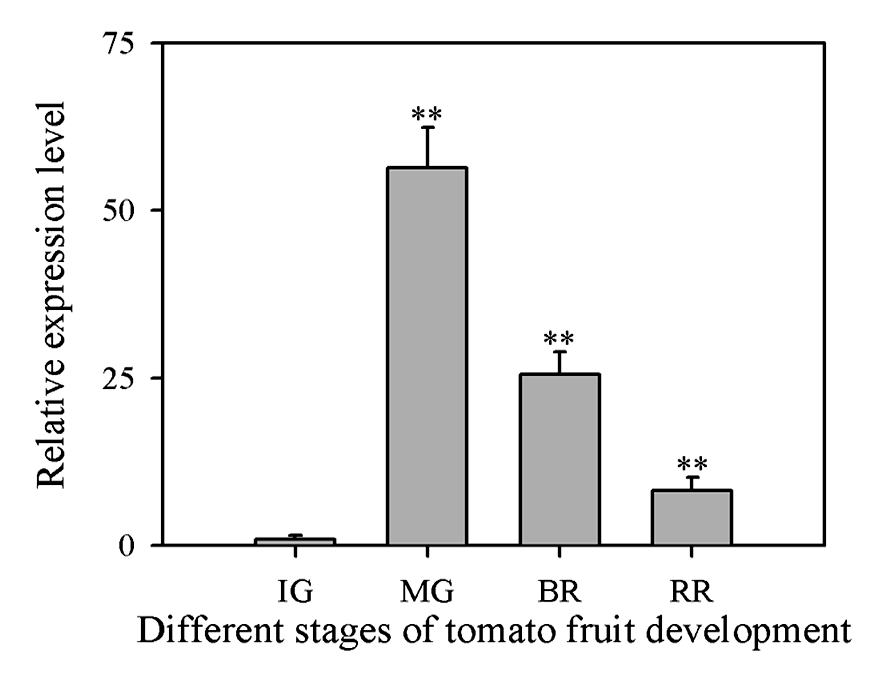

Supplement: Supplementary file 2 [file Image_2.TIF]
