# Supplementary material for: Soluble Starch Synthase III-1 in Amylopectin Metabolism of Banana Fruit: Characterization, Expression, Enzyme Activity, and Functional Analyses
Source: Front Plant Sci. 2017 Mar 30;8:454. doi: 10.3389/fpls.2017.00454 (PMC5371607; doi:10.3389/fpls.2017.00454)
Supplement: Supplementary file 3 [file Table_1.DOC]

**Table S1. Primers used in this study**

| Gene name | Forward primer (5′-3′) | Reverse primer (5′-3′) |
| --- | --- | --- |
| *MaSSI* | CCCATGGGATGGCGGCGATCTGC | GACTAGTCTTATCTGATGTATGGAG |
| *MaSSII* | CCCATGGGATGAAGGGAAATCCC | GACTAGTCTCACCATTGGTACTTG |
| *MaSSIII-1* | CCCATGGGATGTTCCGTGTTTCA | GACTAGTCTTATGATACGTGCCTG |
| *MaSSIII-2* | CCCATGGGATGGCTCTCCAGCC | GACTAGTCCTAAGAATACAGAACC |
| *MaSSI*-qPCR | AGCAGTGGTCAGGGAACAGGGT | ATTGTATCGCAGCACTGTCCCA |
| *MaSSII*-qPCR | GTATGGGACTGTGCCCGTTGTG | TTCAGACAATGACCCAGTGCCT |
| *MaSSIII-1*-qPCR | CAGACTTCATTCTTGTTCCATC | TGCCTGTTGAGAGCATAGTCCA |
| *MaSSIII-2*-qPCR | ATGGAGCAAGACTGGTCGTGGA | AGAATACAGAACCAGAGGAAAC |
| *MaACTIN*  *MaGAPDH*  *SIACTIN* | CAGTGGTCGTACAACTGGTAT  GCAAGATGCCCCAATGT  GTCCTCTTCCAGCCATCCAT | ATCCTCCAATCCAGACACTGT  AGCAAGACAGTTGGTTGTGCAG  ACCACTGAGCACAATGTTACCG |
