# Supplementary material for: Soluble Starch Synthase III-1 in Amylopectin Metabolism of Banana Fruit: Characterization, Expression, Enzyme Activity, and Functional Analyses
Source: Front Plant Sci. 2017 Mar 30;8:454. doi: 10.3389/fpls.2017.00454 (PMC5371607; doi:10.3389/fpls.2017.00454)
Supplement: Supplementary file 4 [file Table_2.DOC]

| MaSSI | MAAICVHRTPFAASKPQPPHGGPRPSPPSTVGSPRWIRRSYCSHVARWWSAAERRSRKEE |
| --- | --- |
| MaSSII | ----------------------------MKGNPPGAIRRSSSAEGQ------EDSDKDDE |
|  |  |
| MaSSI | DWPYLSAIGKAADPDGGAVQENGLLDPTLLAIGRTQNLGDDSVGEQKTEELAGVLEEEET |
| MaSSII | DITDDALRATIKQSKKVLEIQKNLLRQIVERRKFVSSIKDNFVTKE--EETVSYNQNYSS |
|  |  |
| MaSSI | NGDDSVSASRTVLDSSVEVSDSSTVPDCIVEETKAEKEVLMSSSALDVEEEEETDEQEQT |
| MaSSII | FSNMDIDNDEDFDRTNNPWKSEYYTMNDEILEVSAEEGMQGSNEQQEVPPEEGIMDSAPL |
|  | **Domain I** |
| MaSSI | QTRVTRTIVFVSAEAAPYSK-TGGLGDVCGSLPISLAARGHRVMVVSPRYLNGVSNKNFA |
| MaSSII | AGPNVMNVIVVAAECAPWCLPVGGLGDVVGALPKSLARRGHRVMVVAPRYGNYAEPKEVG |
|  |  |
| MaSSI | NAMILESASRFCALEENMKFLFIMSTEQVFVDHPSYHRAGNPYGDSNGAFGDNQFRFTIL |
| MaSSII | VRKRYKVDG--QDMEVMYYHAFIDRVDFVFIDSPVFRHIGN--DIYGGNRLDILKRMILF |
|  |  |
| MaSSI | CYAACEAPLVLPLGGYTYGE-KSLFIVNDWHASLVAVLLAAKYRPYGVYKDARSILVIHN |
| MaSSII | CKAAVEVPWHVPCGGTCYGDGNLVFVANDWHTSLLPVYLKAYYRDNGLMIYARCVLVIHN |
|  |  |
| MaSSI | LAHQGVEPATTYEYMGLPPEWYGALEWIFPTWARKHALDKGEAVNLLKGAIVTADRIVTV |
| MaSSII | IAHQGRGPINDFSYVDLPGHYMDFFKLYDPVGG--------EHFNIFAAGLKAADRLVTV |
|  |  |
| MaSSI | SQGYSWEITTPEGGHGLNELLNSRKFVLNGITNGIDTNEWNPTSDKHIP----FHYSVDD |
| MaSSII | SHGYAWELKTPEGGWGLHGIINDNDWKFQGIVNGIDTRSWNPKFDMYLQSDGYTNYSLET |
|  |  |
| MaSSI | LSG-KAQCKAALQKELGLPIRPDCPLIGFIGRLDYQKGTDVIRSALHELLQDDIQFIMLG |
| MaSSII | LQMGKSQCKAALQ--------------------------HLIADAMSWLVDQDLQLIMLG |
|  | **Domain II** |
| MaSSI | SGNPETEDWMRWVESTNREKFRGWVGFNVPVSHRITAGCDILLMPSRYEPCGLNQLYAMR |
| MaSSII | TGRPDLEDMLRKFESEHHDKVRGWVGFSVKMAHRFTAGADVLLMPSRFEPCGLNQLYAMM |
|  | **Domain III** |
| MaSSI | YGTVPVVNCTGGLRDTVENFDPFATDSSGQGTGWRFSPLSKESMLLKLRVAIQTYREHKA |
| MaSSII | YGTVPVVHAVGGLRDTVKQFDPFN----ETGLGWTFKRAEANKMIEALGHCLNTYRNYKD |
|  |  |
| MaSSI | SWEGLMKRGMSKDFSWDSAAIQYERIFDWTFVDPPYIR |
| MaSSII | SWEGLQRRGMMQDLSWDNAAQRYEDVLVAAKYQW---- |

**Table S2.**
