# Supplementary material for: Soluble Starch Synthase III-1 in Amylopectin Metabolism of Banana Fruit: Characterization, Expression, Enzyme Activity, and Functional Analyses
Source: Front Plant Sci. 2017 Mar 30;8:454. doi: 10.3389/fpls.2017.00454 (PMC5371607; doi:10.3389/fpls.2017.00454)
Supplement: Supplementary file 5 [file Table_3.DOC]

|  | **Transit peptide region** |
| --- | --- |
| MaSSIII-1 | **MSLQPQRPICCPRAVPEHTRFRIRPVHGLFSHSTLGFREVTRIGTTPCMLASSGQWFLCH** |
| MaSSIII-2 | **MALQPQRPVCCPGTVSENSHLRIRSVLGLFSQSSLRFQEVTRISATPRMLAGSG------** |
|  |  |
| MaSSIII-1 | **LKSRTSLSMCNLHVFELHSAGRRPRRTLTPGTKASTRKGFAPRPQVGTSTQKKDQNDAQE** |
| MaSSIII-2 | **------------------YSKRQPRRSLIPMTKASNPRGFAPKPQAGTSIRKKDQNKVDE** |
|  | **Variable repeat region** |
| MaSSIII-1 | **EEVSGSSGSKQPAISSSGTTDRKVTE---EKKLAVVESSLDISDVEELAEAHDSTENAVL** |
| MaSSIII-2 | **KEVSGSSSSSKHTISSSGLTDRRVAAGEKETKVAVSESNLGIIDVDEPPEDQNGIAYTTT** |
|  |  |
| MaSSIII-1 | **DMEESLLKQKADMEAKAQRQLLENLADENFSEGIKVFVVPQVVNPDQVIEIFFNRSLSAL** |
| MaSSIII-2 | **AIQESLVKLKADMDARAHKQLLENLADQNFSRGNKVFVVPQIVNPDQVIKVFLNRSSSAL** |
|  | **SSIII specific region** |
| MaSSIII-1 | A**NEPDVLIK**GAYNGWRWQFFTEKLQKTDLKGDWWSCRLSVPKEAYKVDFVFFNGADAYEN |
| MaSSIII-2 | A**NEADVLIK**GAYNGWRWKFFTEKLQKADIKGDWWSCQIYVPKEAYRVDYVFFNGANTYEN |
|  |  |
| MaSSIII-1 | NNSKDFSLPVE-------------------------EQAEKERQAEEHRQKAAEKAAKSA |
| MaSSIII-2 | NNSEDFFLLVEGGMDEVAFEDFLLEEEHKKLKKLAAEQAEKERQAKEQRRKEAEKVASEA |
|  |  |
| MaSSIII-1 | S---------------------------HIWQIEPNFFEGGDRVRLYYNRSSRPLAHATE |
| MaSSIII-2 | DRAQAKVEVEKKKRGFNHVMKLASNSAHHIWHIEPSLFKGGDRVRLYYNRSCRPLSHAAE |
|  |  |
| MaSSIII-1 | IWIHGGHNIWSEGLSIIEKLSHSEKRDGDWWSADVVVPDRALVLDWVFADGPPGKAVVYD |
| MaSSIII-2 | IWIHGGFNNWDEGLSIIEKLSHSEKRDGDWWFADVAVPGRALVLDWVFADGPPGKAIVYD |
|  |  |
| MaSSIII-1 | NNNRQDFHATVPKSMPGELFWVEEEHRIYRKLQEERRAREEAVHKKAEKTARMKAETKEK |
| MaSSIII-2 | NNNLQDFHATVPKSIPVEVSWVEEENQIYRKFQEDRRSKEEAIRKKTEKTVRMKAETKER |
|  |  |
| MaSSIII-1 | TMKMFLLSQKHIVYTEPIDVRAGSVITVLYNPSNTVLNGKPEVWFRCSFNRWSHHNGPLP |
| MaSSIII-2 | TMKLFLLSQKHIVYSEPIVVRAGRMVTVFYNPYHTVLNGKPEVWFKCSFNRWNHRKGPLP |
|  |  |
| MaSSIII-1 | PQKMVPAENASHLKATVKVPMDAYMMDFVFSEREDGGIYDNRNGMDYHIPVIGGIAKEPP |
| MaSSIII-2 | PQKMVPAENGSHLKATVKVPMDAYMMDFVFSERENGGIYDNRNRMDYHIPVTGGIAKEPP |
|  | **Catalytic Domain** |
| MaSSIII-1 | MHIVHIAVEMA**PIAKVGGLGDVVTSLS**RAVQDLGHTVDVVLPKYNCMNLSNVKDLHFRNS |
| MaSSIII-2 | MHIVHIAVEMA**PIAKVGGLGDVVTSLS**RAVQDLGHTVVVVLPKYDCMNLSNVKDLHHRNS |
|  |  |
| MaSSIII-1 | FAWDGMEINVWFGQVEGLPVYFLEPQNGMFSVGCIYGRNDDGHRFGFFCHAALEFLLQSG |
| MaSSIII-2 | FAWGETEISVWFGKVEGLPVYFLEPKNGMFSVGCIYGRSDDGHRFGFFCHAALEFLLQSG |
|  |  |
| MaSSIII-1 | FQPDILHCHDWSSAPVAWLFKEHYAHYGLSNARVIFTIHNLEFGVHNIGRAMAYADKATT |
| MaSSIII-2 | FRPDILHCHDWSSAPVAWLFKEHYIYYGLSNARVIFTIHNLEFGVHNIGRAMTYADKATT |
|  |  |
| MaSSIII-1 | DLWLFDIQFPFRW----------------------------------------------- |
| MaSSIII-2 | VSQTYSREVAGNPAISPHLHKFHGIVNGIDPDIWDPYNDQFIPVPYTPENVVEGKKAAKE |
|  |  |
| MaSSIII-1 | ------------------------------------------------------------ |
| MaSSIII-2 | ALQQKLGLKTSDHPLVGIITRLTVQKGIHLIKHAVWRTLEHNGQVVLLGSAPDSRIQNDF |
|  |  |
| MaSSIII-1 | ------------------------------------------------------------ |
| MaSSIII-2 | ANLASELHSSHGDRVRFCLTYNEPLSHLIYAGADFILVPSLFEPCGLTQLIAMRYGSIPV |
|  |  |
| MaSSIII-1 | ------------------------------------------------------------ |
| MaSSIII-2 | VRRTGGLYDTVFDVDTERERAQAQGLEPNGFSFDGADSADVDDVLNRAISAWFDKREWFH |
|  |  |
| MaSSIII-1 | ----------------------------------- |
| MaSSIII-2 | SLCKRVMEQDWSWNRPALDYMELYHSARNKTS |

**Table S3.**
